# Supplementary material for: 18F‐AV‐1451 and CSF T‐tau and P‐tau as biomarkers in Alzheimer's disease
Source: EMBO Mol Med. 2017 Jul 25;9(9):1212–23. doi: 10.15252/emmm.201707809 (PMC5582410; doi:10.15252/emmm.201707809)
Supplement: Supplementary file 1 — Expanded View Figures PDF [file EMMM-9-1212-s001.pdf]

Expanded View Figures

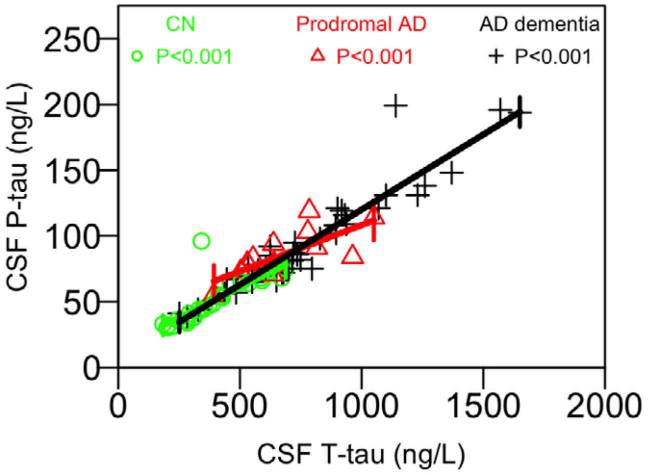

Figure EV1. Correlations between CSF T-tau and P-tau.

CSF P-tau and T-tau in different diagnostic groups, with slopes from linear regression models adjusted for age and sex.

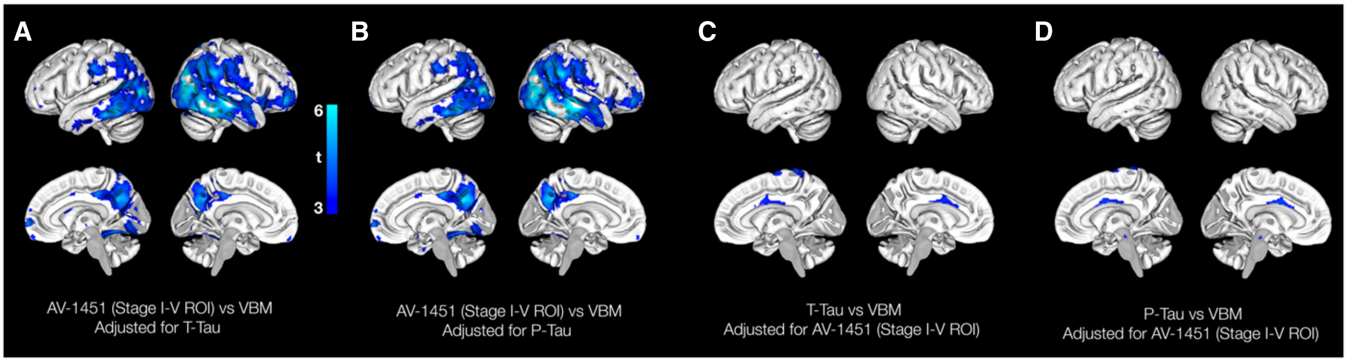

Figure EV2. Associations between atrophy and CSF and PET tau, adjusted for the other tau modality.

A, B VBM analyses for ^{18}F-AV-1451 when adjusted for CSF T-tau or P-tau.
C, D VBM analyses for CSF T-tau and P-tau when adjusted for ^{18}F-AV-1451.

© 2017 The Authors

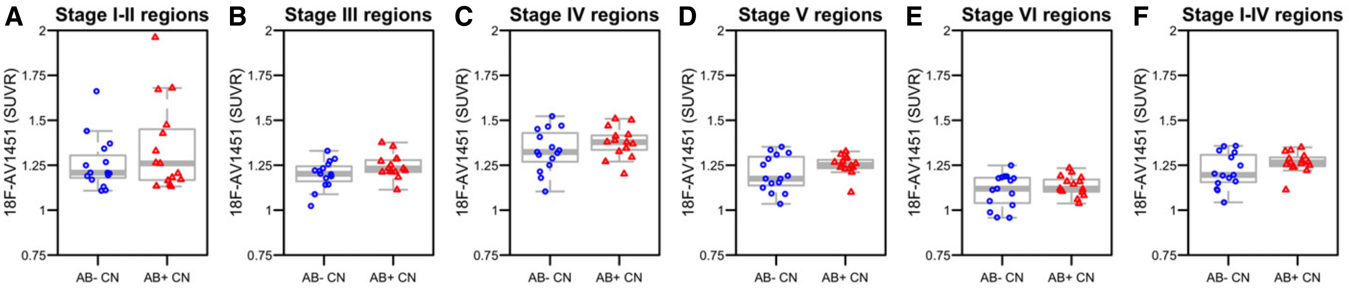

Figure EV3. Tau PET in preclinical AD.

A–F Regional ^{18}F-AV-1451 by Aβ-status in controls. The boxplots show medians and interquartile ranges. There were no significant differences between Aβ-negative and Aβ-positive controls (tau stage I–II region, *P* = 0.53; tau stage III region, *P* = 0.19; tau stage IV region, *P* = 0.63; tau stage V region, *P* = 0.30; tau stage VI region, *P* = 0.72; and tau stage I–V composite region, *P* = 0.31). Tests were done by linear regression models adjusted for age and sex.

 © 2017 The Authors
