# Supplementary material for: 18F‐AV‐1451 and CSF T‐tau and P‐tau as biomarkers in Alzheimer's disease
Source: EMBO Mol Med. 2017 Jul 25;9(9):1212–23. doi: 10.15252/emmm.201707809 (PMC5582410; doi:10.15252/emmm.201707809)
Supplement: Supplementary file 2 — Table EV1 [file EMMM-9-1212-s002.docx]

**Table EV1 Associations between CSF tau and regional 18F-AV-1451 retention**

| **CSF biomarker** | **^18^F-AV-1451 tau stage region** | **Statistics** | **Controls** | **Prodromal AD** | **AD dementia** |
| --- | --- | --- | --- | --- | --- |
| T-tau | I-II | β | 0.000215 | 0.00111 | 0.000141 |
|  |  | p-value | 0.621 | 0.0386 | 0.451 |
|  | III | β | 0.000368 | 0.000562 | 0.000784 |
|  |  | p-value | 0.517 | 0.417 | <0.01 |
|  | IV | β | 0.000734 | 0.000729 | 0.000579 |
|  |  | p-value | 0.34 | 0.437 | 0.0819 |
|  | V | β | 0.000608 | -0.00014 | 0.000862 |
|  |  | p-value | 0.218 | 0.816 | <0.001 |
|  | VI | β | 0.000285 | 0.0000782 | 0.000383 |
|  |  | p-value | 0.525 | 0.886 | 0.0496 |
|  | I-V composite | β | 0.0006 | 0.0000148 | 0.00081 |
|  |  | p-value | 0.229 | 0.981 | <0.001 |
| P-tau | I-II | β | 0.00147 | 0.00447 | 0.00158 |
|  |  | p-value | 0.718 | 0.447 | 0.313 |
|  | III | β | 0.00255 | -0.00186 | 0.00567 |
|  |  | p-value | 0.63 | 0.808 | <0.01 |
|  | IV | β | 0.00667 | -0.0145 | 0.00353 |
|  |  | p-value | 0.342 | 0.154 | 0.193 |
|  | V | β | 0.00614 | -0.00913 | 0.00606 |
|  |  | p-value | 0.180 | 0.168 | <0.001 |
|  | VI | β | 0.00297 | -0.000827 | 0.003 |
|  |  | p-value | 0.469 | 0.889 | 0.0601 |
|  | I-V composite | β | 0.00591 | -0.00897 | 0.00567 |
|  |  | p-value | 0.201 | 0.179 | <0.01 |

β-coefficients and P-values are from the linear regression models visualized in Figure 1.
